# Household cluster of psittacosis caused by *Chlamydia psittaci* ST388 in China: a case report and genomic analysis

**DOI:** 10.1186/s12879-026-13940-0

**Published:** 2026-07-21

**Authors:** Yuanhang Wang, Xiaofei Fu, Yang Liu, Rui Li, Guoying Zhu, Zhongwen Chen

**Affiliations:** Jiaxing Center for Disease Control and Prevention, Jiaxing, China

**Keywords:** Psittacosis, Household cluster, *Chlamydia psittaci*, ST388, Genomic epidemiology

## Abstract

In March 2025, a married couple in Jiaxing City, Zhejiang Province, China, presented with fever and pneumonia. Metagenomic next-generation sequencing (mNGS) of bronchoalveolar lavage fluid confirmed *Chlamydia psittaci* (*C. psittaci*) infection. Epidemiological investigation revealed exposure to a pet budgerigar purchased one month prior. Environmental sampling detected *C. psittaci* in the bird’s feces and the patients’ living spaces. Phylogenetic analysis of the *ompA* gene identified the strains as genotype A, and multilocus sequence typing (MLST) determined the sequence type (ST) as ST388. Two cohabiting elderly parents without bird exposure remained asymptomatic and PCR-negative. Phylogenetic analysis showed the avian and environmental isolates formed a distinct clonal cluster. This report highlights the risk of zoonotic transmission from asymptomatic avian carriers and the utility of genomic surveillance in outbreak investigation. Clinicians should consider psittacosis in atypical pneumonia cases with bird exposure.

## Background

Psittacosis, caused by *Chlamydia psittaci* (*C. psittaci*), is a zoonosis often presenting as atypical pneumonia [1–2]. While sporadic cases are reported globally, household clusters are relatively scarce, and the molecular epidemiology of circulating strains in China remains poorly characterized. *C. psittaci* comprises multiple genotypes which are predominantly identified through sequencing of the gene encoding *outer membrane protein A* (*ompA*) [3–5]. Genotype A is typically associated with psittacine birds and high virulence [6–7]. Recent advancements in metagenomic sequencing (mNGS) and multilocus sequence typing (MLST) have improved strain characterization [8]. However, transmission dynamics and genomic characteristics of emerging sequence types (ST) in household settings remain rarely reported. We report the household cluster of psittacosis caused by *C. psittaci* ST388 in China, illustrating the value of genomic epidemiology and environmental sampling in transmission source identification.

## Methods

### Data source

Two cases of psittacosis were reported by a hospital in Zhejiang Province through the China Information System for Disease Control and Prevention (CISDCP), specifically under the “Other Diseases” module. Both cases were diagnosed by mNGS of bronchoalveolar lavage fluid (BALF) at a provincial hospital in Hangzhou, Zhejiang Province. Since the diagnosis was made at a hospital outside our municipal jurisdiction, the original sequencing data and clinical isolates were not accessible for genomic analysis in this study.

### Diagnostic criteria

Both cases were diagnosed according to the Chinese Expert Consensus on the Diagnosis and Treatment of Psittacosis [9]. The diagnostic criteria for confirmed psittacosis include: (1) clinical manifestations consistent with influenza-like atypical pneumonia; (2) a history of avian exposure; and (3) detection of *C. psittaci* nucleic acid in respiratory specimens by mNGS. Both patients met all three criteria, confirming *C. psittaci* as the etiological agent.

### Sample collection

Five throat swab samples (two parents and three colleagues) were collected from individuals who had close contact with the patients. Additionally, 21 environmental swab samples were obtained, including 9 samples from the household budgerigar (1 throat, 1 cloaca, 3 feces, 3 cages, 1 feeding trough) and 12 samples from the patients’ residential areas (5 kitchen areas, 7 from patients’ bedroom and bathroom areas).

### DNA extraction and detection

DNA was extracted from the samples using an EXM6000 nucleic acid extractor (Zybio, Chongqing, China) with the corresponding extraction kit (Zybio RB-T 200, Lot.2411002). The DNA was detected by real-time PCR diagnostic kit for rapid detection of *C. psittaci* (D1701YH, x-abt, Beijing, China). Samples showing Ct values below 36 were defined as positive.

Targeted capture sequencing for avian and environmental samples.

For the avian fecal and environmental swab samples, the *C. psittaci* whole genome was enriched from the extracted DNA using the Target Capture Kit for *C. psittaci* (iGeneTech Biotech (Beijing) Co., Ltd., Beijing, China; catalog number PH2002161). For MGI DNBSEQ-T7RS sequencing, a sequencing library was constructed in accordance with the manufacturer’s protocols provided with the library building kit (iGeneTech Biotech (Beijing) Co., Ltd., Beijing, China; catalog number C11361).

### Phylogenetic analysis of the *ompA* gene

The *ompA* gene sequences from three *C. psittaci* strains isolated in this study were extracted and aligned with the downloaded reference sequences using MUSCLE v5.1 [10] with default parameters: muscle -align input.fa -output aln.afa. The resulting multiple sequence alignment was refined using trimAl v1.5.rev0 [11] with the parameters: trimal -in aln.afa -out aln.AUTOtrim.fas -automated1. A phylogenetic tree was constructed from the refined alignment using IQ-TREE v2.4.0 [12] with the parameters: -m MFP -b 1000 -nt AUTO -o AF269282.1. The tree was subsequently visualized using the Interactive Tree Of Life (iTOL) platform (https://itol.embl.de/) [13].

### Phylogenetic analysis of genotype A core genome single nucleotide polymorphism (cgSNP)

A total of 70 complete or near-complete *C. psittaci* genotype A genomes were initially retrieved from public databases. Preliminary phylogenetic analysis identified eight genomes with exceptionally long branch lengths. These eight sequences were excluded as long-branch outliers, and the remaining 62 high-quality genomes were used for the subsequent cgSNP analysis. The genomes of *C. psittaci*, along with the corresponding genotype and host information, were downloaded from the RefSeq and GenBank databases of NCBI on 21 May 2025. For cases where the authors did not provide the genotype, genotyping was carried out based on the *ompA* gene. The unique ST of the genome was determined through standardized MLST analysis of nucleotide variations in multiple housekeeping genes by the *Chlamydiales* database on PubMLST (https://pubmlst.org/) [14]. RhierBAPS 1.1.4 [15] was used to analyze the subpopulation group (SG) of the above genomes and the three genomes (2025-0002-1, 2025-0002-2, 2025-0002-3) in this study, with the following parameters: hierBAPS (snp.matrix, max.depth = 2, n.pops = 20, quiet = TRUE). Using GCF_000204255.1 as the reference genome, Snippy v4.6.0 (https://github.com/tseemann/snippy) was used with default parameters (snippy --reference ref.fa --ctgs assembly1.fa --outdir assembly1; snippy-core --ref ref.fa --prefix core assembly1 assembly2...) to obtain the cgSNP alignment sequences. A phylogenetic tree was constructed from the cgSNP alignment using IQ-TREE v2.4.0 [12] with the following parameters: -s core.aln -m MFP -b 1000 -nt AUTO. The tree was visualized using the R package ggtree 4.0.3 [16] with default parameters: ggtree (tree, layout = “circular”, branch.length = “none”).

## Results

### Case presentation

Case 1, a 43-year-old male presented on 4 March 2025, with fever (39.2 °C), cough, and dizziness. Chest computed tomography (CT) showed left lung inflammation on March 5 and initial therapy with compound paracetamol tablets (II) for fever reduction failed at a county-level hospital in Jiaxing City. He was transferred to a provincial hospital on March 6. mNGS of BALF on March 8 confirmed *C. psittaci*. Case 2, the patient’s 47-year-old wife developed fever (39 °C), cough, and diarrhea on March 9. CT indicated mild pneumonia. BALF mNGS on March 11 confirmed *C. psittaci*. The temporal courses of symptom onset in the two patients are illustrated in Fig. 1.

**Fig. 1:**
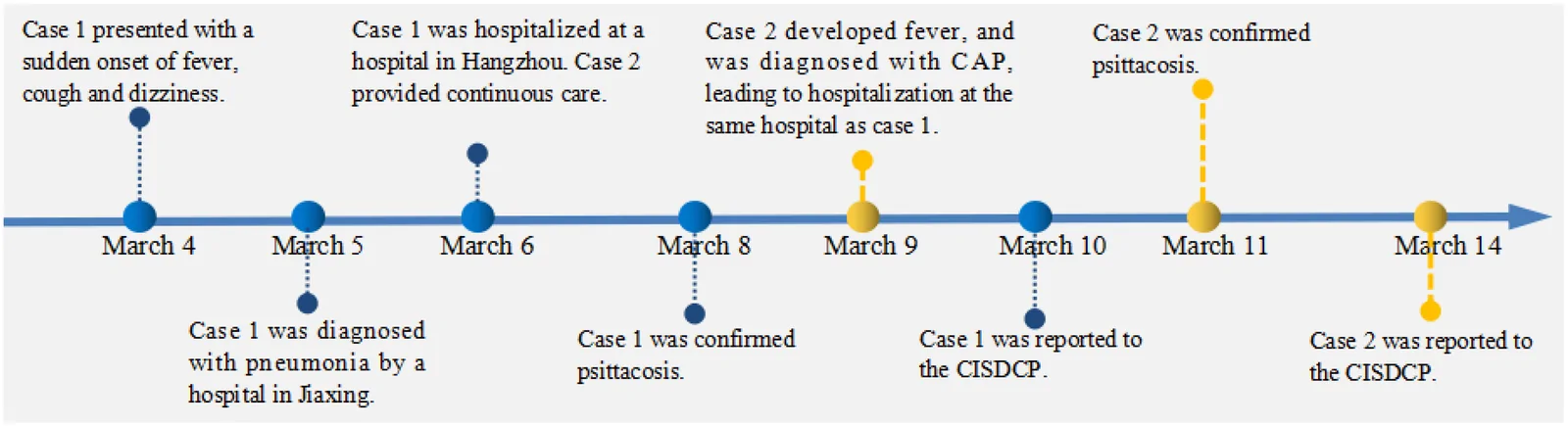
The symptom onset timelines in the two patients. Blue circles indicate events for Case 1, and yellow circles indicate events for Case 2. Case 1 was the first to develop symptoms on 4 March 2025, and Case 2 belonged to the same family. CAP stands for community-acquired pneumonia. CISDCP stands for China Information System for Disease Control and Prevention

### Treatment and outcomes

Upon admission, chest imaging of Case 1 demonstrated bilateral prominent lung markings with patchy high-density opacities in the left lung and absence of pleural effusion (Fig. 2). Abnormal laboratory test results were obtained, as follows: procalcitonin (PCT: 0.33 ng/mL; normal < 0.05 ng/mL), hypersensitive C-reactive protein (hs-CRP: 12.8 mg/L; normal < 0.5 mg/L), monocyte count (MO: 0.77 × 10⁹/L; normal 0.1–0.6 × 10⁹/L), hemoglobin (Hb: 117 g/L; normal 120–150 g/L), and blood platelet count (PLT: 449 × 10⁹/L; normal 125–350 × 10⁹/L). Treatment with omadacycline combined with oxygen nebulization following etiological confirmation resulted in marked clinical improvement, including normalization of body temperature and reduction of inflammatory markers, culminating in discharge with prescribed medications (compound glycyrrhizin tablets and doxycycline capsules) on 10 March 2025. Case 2 received an identical antimicrobial regimen, achieving resolution of fever and attenuated inflammation, though with persistent productive cough, and was discharged with symptomatic medications (compound glycyrrhizin tablets, eucalyptol limonene and pinene enteric soft capsules, moxifloxacin budesonide and formoterol fumarate powder for inhalation and bifidobacterium capsules) on 15 March 2025.

**Fig. 2:**
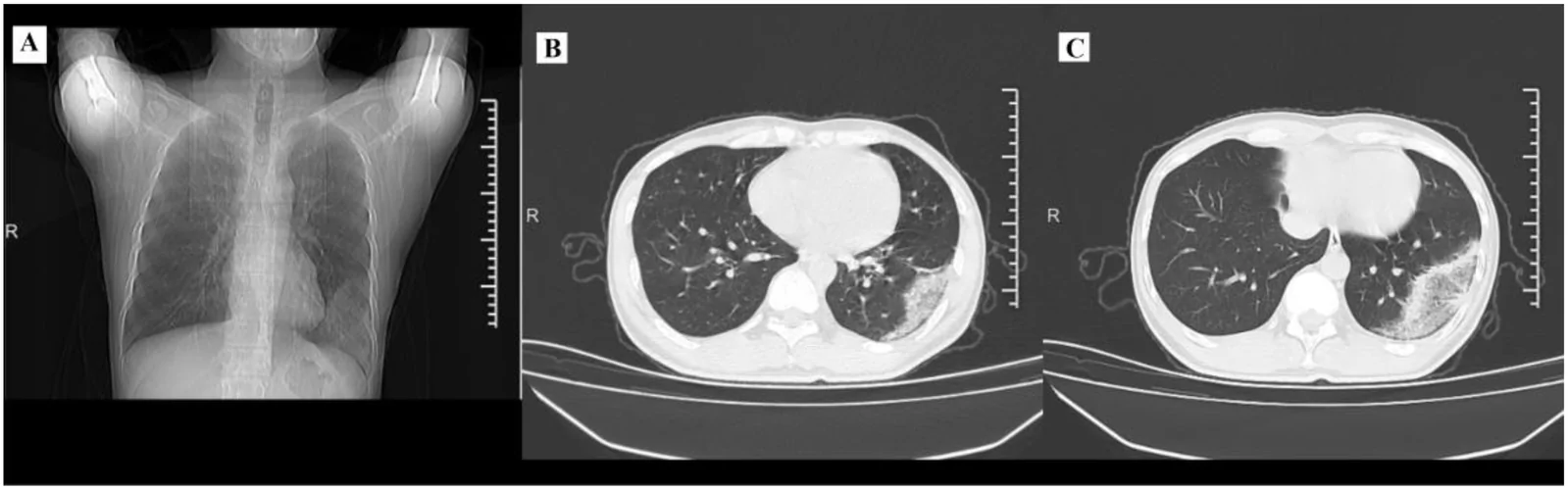
Chest CT imaging findings of Case 1 on 5 March 2025. (**A**) Frontal chest radiograph shows bilateral increased bronchovascular markings and an ill-defined patchy opacity in the left mid-lower lung. (**B**) Axial CT (upper lung window): Bilateral bronchovascular bundle thickening; a wedge-shaped subpleural patchy opacity is present in the left lower lobe, with normal hilar, bronchial and mediastinal structures. (**C**) Axial CT (lower lung window): The left subpleural pulmonary opacity becomes larger; bilateral bronchovascular thickening persists, and no mediastinal lymphadenopathy is identified

### Epidemiological investigation

The couple resided with Case 1’s parents in a rural self-built house (completed in 2022) in Jiaxing. On 23 February 2025, Case 1 purchased a pet budgerigar, which was housed exclusively on the fourth floor with no access to other floors. Case 1’s parents and the couple lived on the second and third floor respectively. Case 1 had daily contact with the budgerigar; Case 2 had occasional exposure. Both cases thus demonstrated definitive psittacine exposure history. Additionally, neither of the two cases had documented exposure to other suspected animals, positive environmental sources, or confirmed psittacosis patients within 45 days before symptom onset. The parents had no bird contact. All five close contacts (two parents and three colleagues) reported no observed abnormalities (e.g., fever, anorexia, or diarrhea).

### Laboratory results

Real-time PCR detected *C. psittaci* in the bird’s feces (Ct = 30) and environmental swabs from the patients’ bathroom (Ct = 32) and bedroom (Ct = 33). Pharyngeal swabs from the parents and three colleagues were negative. Phylogenetic analysis of the *ompA* gene classified all isolates as genotype A (Fig. 3A). CgSNP analysis of 62 Type A genomes revealed the three avian and environmental isolates formed a tight monophyletic cluster belonging to ST388 (Fig. 3B). This ST was recently deposited in PubMLST by the Zhejiang Provincial Center for Disease Control and Prevention (Zhejiang CDC) [17], suggesting ST388 may be an emerging sequence type in the region.

**Fig. 3 A:**
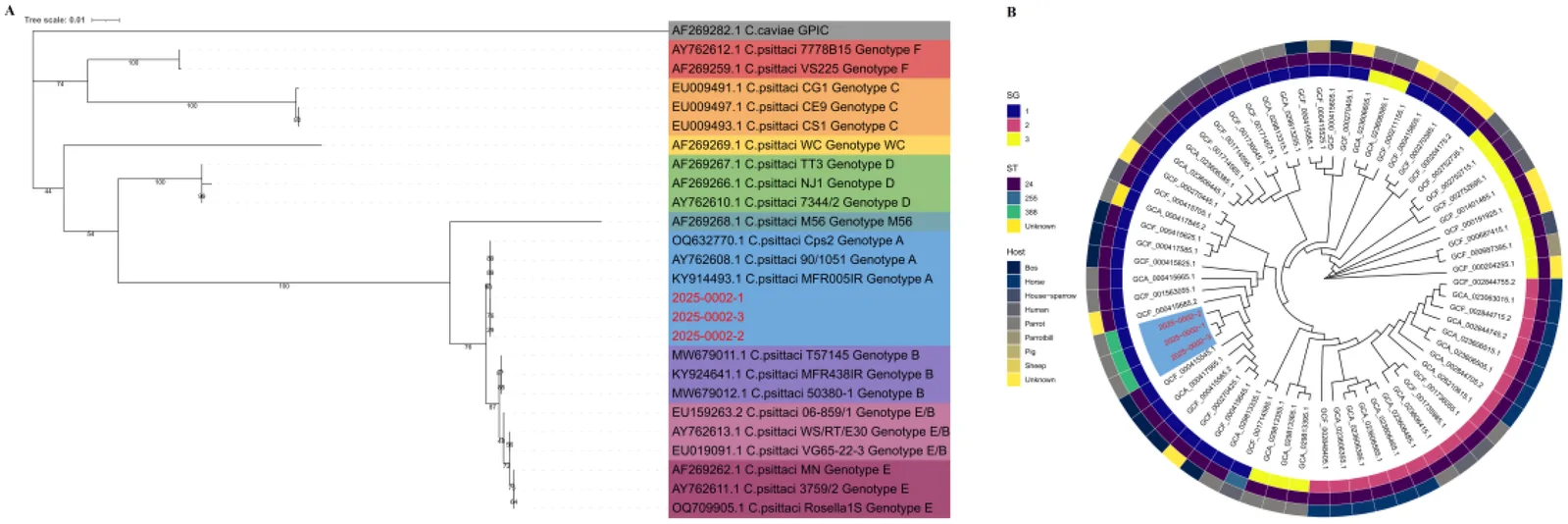
Phylogenetic tree of *C. psittaci ompA* gene sequences from the study isolates and representative genotypes. 23 reference sequences, comprising the *ompA* genes of major *C. psittaci* genotypes (A, B, C, D, E, F, E/B, WC, M56) and *Chlamydia trachomatis* AF269282.1 (outgroup, gray branch), were retrieved from the NCBI Nucleotide database. Phylogenetic analysis classified the three avian and environmental isolates in this study as genotype A. Figure 3B Phylogenetic tree analysis of *C. psittaci* genotype A based on cgSNP. The GCF_000204255.1 recommended by the NCBI RefSeq database was employed as the reference genome. SG indicates population structure; ST indicates sequence type; and host indicates the source organisms of each genome. The three isolates from the present study, namely 2025-0002-1 (patient’s bathroom, environmental sample), 2025-0002-2 (parrot feces, avian source), and 2025-0002-3 (patient’s bedroom, environmental sample), are highlighted in red font

## Discussion and conclusions

In light of the escalating human-animal interface resulting from global urbanization and climate change, psittacosis, an easily overlooked public health concern, warrants significant attention. Recent studies have indicated that the rising population of domestic pet birds, in conjunction with increased interactions between wild and captive avian species, has heightened the risk of *C. psittaci* transmission, thereby contributing to a rise in the incidence of human infections [18–19]. A comprehensive understanding of the clinical manifestations and molecular epidemiology of *C. psittaci* is crucial for advancing therapeutic interventions and preventive strategies.

This study documents a household cluster of *C. psittaci* ST388 (genotype A) in China. Epidemiological investigation confirmed that both patients had direct contact with a pet parrot, which remained asymptomatic throughout the period under analysis. No other suspected sources of infection were identified. These findings suggest that the parrot may have been the source of infection in this household cluster, which prompted the investigation presented in this study.

The incubation period of psittacosis ranges from 5 to 14 days (range 3–45 days). The delayed onset of Case 2 relative to Case 1 can be attributed to variable incubation periods following independent exposure to the common source, the pet parrot. This conclusion is supported by negative PCR results from pharyngeal swabs of close contacts (parents and workplace colleagues) and negative environmental swabs from shared living spaces (kitchen). These combined findings indicate that human-to-human transmission was unlikely.

Phylogenetic analysis of the *ompA* gene classified all three isolates (one avian and two environmental) as genotype A, the dominant zoonotic genotype associated with human infections worldwide. cgSNP analysis further demonstrated that the three isolates from parrot feces, the patient’s bathroom, and the patient’s bedroom formed a tight, distinct cluster belonging to ST388, a novel sequence type recently deposited in PubMLST by Zhejiang CDC. The high genetic homology among these avian and environmental isolates confirms they originated from the same clonal strain, providing robust genomic evidence for the bird–environment–human transmission route. While genotype A is common globally, the specific prevalence of ST388 in Zhejiang warrants further surveillance.

Regarding the route of environmental contamination, parrot feces were the most likely source. Since the bird had no direct access to the patients’ bedroom and bathroom, the detection of genetically identical *C. psittaci* ST388 strains in these areas provides strong evidence for fomite-mediated contamination. Specifically, the patients most likely carried the pathogen from the bird’s living area to their own rooms via contaminated clothing or personal items. The relatively low detection rate in the avian-associated samples is attributable to intermittent shedding of C. psittaci in birds, a recognized cause of false-negative results in single-time-point sampling [20–21]. In contrast, contamination may have accumulated in patients’ living spaces via repeated fomite carriage, explaining the comparable detection frequency. Notably, environmental swabs from the kitchen, located on the first floor, a common area used by all household members, were negative by PCR. This spatial pattern, positivity in the patients’ bedroom and bathroom but negativity in the kitchen, suggests that contamination was introduced into specific areas through fomite carriage by the patients, rather than through airborne dispersion.

Diagnosis was delayed initially due to non-specific symptoms resembling community-acquired pneumonia, a common challenge in primary care. mNGS was critical for definitive diagnosis. Both patients responded well to omadacycline, consistent with guidelines recommending tetracyclines. The absence of apparent illness in the pet parrot, despite confirmed shedding of *C. psittaci*, highlights the public health risk posed by asymptomatic carriers in the pet trade.

Several limitations should be acknowledged. First, the clinical diagnosis of both cases was established by mNGS at a hospital located outside Jiaxing City. The original mNGS sequencing data and clinical isolates were not accessible, precluding direct genomic comparison between the human clinical strains and the avian/environmental isolates. Second, the sample size is inherently limited by the nature of a household cluster case report, which restricts the generalizability of the findings. Despite these limitations, this study demonstrates the value of environmental genomic surveillance within the One Health framework for tracking the transmission of emerging *C. psittaci* genotypes. Future prospective studies should aim to include concurrent sampling and genomic analysis of clinical, animal, and environmental specimens to enable direct comparisons and further strengthen transmission chain evidence.

In conclusion, this cluster highlights the need for heightened clinician awareness of psittacosis in atypical pneumonia cases and the value of environmental genomic surveillance in identifying transmission chains under the “One Health” framework to track emerging *C. psittaci* genotypes.

## Data Availability

The datasets generated and analysed during the current study are available in the National Center for Biotechnology Information and registered as BioProject number PRJNA1441873.
